# Inflammasome-Independent Role of NLRP3 Mediates Mitochondrial Regulation in Renal Injury

**DOI:** 10.3389/fimmu.2018.02563

**Published:** 2018-11-12

**Authors:** Su-Mi Kim, Yang Gyun Kim, Dong-Jin Kim, Seon Hwa Park, Kyung-Hwan Jeong, Yu Ho Lee, Sung Jig Lim, Sang-Ho Lee, Ju-Young Moon

**Affiliations:** ^1^Division of Nephrology, Department of Internal Medicine, Kyung Hee University, College of Medicine, Seoul, South Korea; ^2^Department of Pathology, Kyung Hee University College of Medicine, Seoul, South Korea; ^3^Kyung Hee Medical Science Research Institute, Kyung Hee University, Seoul, South Korea

**Keywords:** NLRP3, mitochondrial ROS, apoptosis, mitophagy, acute kidney injury (AKI)

## Abstract

The NOD-like receptor family, pyrin domain containing-3 (NLRP3) inflammasome has been implicated in renal inflammation and fibrosis. However, the biological function of inflammasome-independent NLRP3 in non-immune cells is still unclear. We evaluated the role of inflammasome-independent NLRP3 in renal tubular cells and assessed the value of NLRP3 as a therapeutic target for acute kidney injury (AKI). Various renal tubular cell lines and primary cultured tubular cells from NLRP3 knockout (KO) mice were used for *in vitro* studies. We also tested the role of tubular NLRP3 in AKI with a unilateral ureter obstruction model (UUO). Hypoxia induced significant increase of NLRP3 independent of ASC, caspase-1, and IL-1β. NLRP3 in renal tubular cells relocalized from the cytosol to the mitochondria during hypoxia and bound to mitochondrial antiviral signal protein **(**MAVS). The deletion of NLRP3 or MAVS in renal tubular cells attenuated mitochondrial reactive oxygen species (ROS) production and depolarization of the mitochondrial membrane potentials under hypoxia. In response to UUO, NLRP3 KO mice showed less fibrosis, apoptosis, and ROS injury than wild type (WT) mice. Compared with WT kidney, mitophagy was up-regulated in NLRP3 KO kidney relative to the baseline and it was protective against AKI. Our results indicate that inflammasome-independent NLRP3 in renal tubular cells plays important role in mitochondrial ROS production and injury by binding to MAVS after hypoxic injury. This mitochondrial regulation in the absence of NLRP3 increases autophagy and attenuates apoptosis after UUO. We suggest that inflammasome-independent NLRP3 could be a therapeutic target of AKI to prevent the progression of chronic kidney disease.

## Introduction

The NOD-like receptor family, pyrin domain-containing-3 (NLRP3) inflammasome is a multi-protein complex and sensor in innate immune cells activated by damage-associated molecular patterns (DAMPs) and it promotes the secretion of pro-inflammatory cytokines such as IL-1β and IL-18. The NLRP3 inflammasome is activated in various kidney diseases both acute and chronic kidney disease, such as ischemic-reperfusion injury (IRI), crystal-induced fibrosis and diabetic nephropathy. The inhibition of the NLRP3 inflammasome by inhibitors or genetic deletion of NLRP3 can ameliorate renal diseases (1–3).Activation of NLRP3 after priming requires the secondary signals such as NLRP3 relocalization to mitochondria, production of mitochondrial reactive oxygen species (ROS), K^+^ efflux, and cathepsin release from lysosomal membranes (4–6). The relocalization of NLRP3 to the mitochondria and mitochondrial ROS and mitochondrial DNA are the major secondary signals in kidney disease (7, 8). We previously reported that mitochondrial ROS is crucial for the activation of the NLRP3 inflammasome in macrophages by hyperuricemia in type 2 diabetic nephropathy and the mitochondrially targeted antioxidant, Mito-TEMPO, attenuated NLRP3 increase, and IL-1β release (9). Intriguingly, the role of inflammasome-independent NLRP3 in non-immune cells also has been reported to be associated with mitochondrial function. Renal tubular epithelial cells express a considerable amount of inflammasome components such as NLRP3, ASC and caspase-1 but do not produce IL-1β (10). NLRP3 colocalizes with cytochrome c and MitoTracker Red in human proximal tubular epithelial cells (11). One study indicated a possibility of renal protective effect of NLRP3 knockout (KO) mice with IRI, which could be associated with inflammasome-independent NLRP3 (12). Additionally, the level of mitochondrial ROS was reduced in NLRP3 KO cardiac fibroblasts, although caspase-1 KO cardiac fibroblasts displayed similar profiles to wild type (WT) cells. NLRP3 deficiency showed protective effects against kidney injury and played similar effect as a ROS scavenger (13, 14). Although inflammasome-independent NLRP3 has been suggested to be related with mitochondrial injury in non-immune cells, the role of inflammasome-independent NLRP3 in kidney disease has not been clarified yet.

Hypoxia has been recognized a major player in acute kidney injury (AKI) and its progression to chronic kidney disease (CKD) (15, 16). The clinical manifestations of renal ischemic injury range from minimal changes in renal function to renal failure requiring renal replacement therapy. Ischemic renal injury predisposing CKD contributes to increase morbidity and mortality. Hypoxia, cellular redox state and mitochondrial function are known to interact with each other in a complicated manner. Mitochondrial fragmentation has been reported to be an early indicator of AKI, and pharmacological inhibitor of mitochondrial fission has shown protective effect in mouse models of ischemic AKI (17, 18). Therefore, targeted therapy has been suggested for protecting mitochondria in ischemic AKI as an ideal strategy for preventing to CKD progression.

We designed this study to clarify the mechanism of inflammasome-independent NLRP3 associated with mitochondrial injury in renal tubular epithelial cells. We also evaluated whether the regulation of NLRP3 in the kidney could prevent AKI and examined the possibility of NLRP3 as the therapeutic target of AKI.

## Materials and methods

### Animal study

RGEM/Cas9 NLRP3 Knockout mice was established from Macrogen (Seoul, Korea). Target RGEM site was located in exon 2 of NLRP3 gene and four target sequences was used (RG1 (down) AGCAGCCCTTTCGAGGGTCTCGG, RG2 (up) GACGAGTGTCCGTTGC AAGC TGG, RG3 (up) CATGATTGACTTCAATGGCGAGG, RG4 (up) GGATCTTTGCTGCG ATC AACAGG). Six kinds of NLRP3 Knock out mutant mice were obtained and confirmed using genotyping with NLRP3 PCR primers. After identification of KO deletion type, mice line, which has large deletion site (>200 bp) of NLRP3, was selected. Male WT and NLRP3 KO mice (NLRP3^−/−^) (aged 8–10 weeks; *n* = 6 per group) underwent left ureteral ligation as described previously (19). Complete ureteral obstruction was produced by double ligation with 4-0 silk thread. These mice comprised the WT/UUO and NLRP3^−/−^/UUO groups, respectively. Comparable groups of male mice underwent an identical procedure without ligation as the WT/Sham group and NLRP3^−/−^/Sham groups (*n* = 6 per group). Mice were sacrificed on days 7 after UUO and the serum and kidney were collected. The level of blood urea nitrogen (BUN) and serum creatinine was determined using a VetTest 8008 (IDEXX Laboratories, Westbrook, ME, USA). All experiments were performed according to the guidelines of the hospital animal research ethics committee (KHNCM AP 2018-004).

### Cell culture

Human kidney-2 (HK-2) cells (human kidney proximal tubular epithelial cell line) were purchased from KCLB (the Korean cell line bank, Seoul, Korea). RPMI1640 was supplemented with 5% fetal bovine serum (FBS) and 1% penicillin/streptomycin (P/S) (WelGENE, Daegu, Republic of Korea). For hypoxic cell culture, cells were placed in a hypoxic (1% O_2_, 5% CO_2_, 37°C) incubator (Ruskinn, Bridgend, UK) for 0, 1, 3, 6, 9, 12, 24, and 48h. Control cells (normoxic cells) were incubated for equivalent periods under normoxic conditions (21% O_2_, 5% CO_2_, 37°C). Transient transfection of NLRP3-pCMV6 vector (ORIGENE, Rockville, MD) was conducted using the Lipofectamine 2,000 method. LC3-GFP vector was kindly provided by Dr. DH Cho (Kyung Hee University School of East-West Medical Science). The human renal proximal tubular epithelial cell line (HKC8) was obtained from Dr. L. Rausen (Johns Hopkins University, Baltimore, MD) and was maintained in Dulbecco's Modified Eagle Medium supplemented with Ham's F12 medium (DMEM/F12; WelGENE, Daegu, Republic of Korea) DMEM/F12 was supplemented with 5% FBS and 1% (P/S). RPTEC/TERT1 cells were purchased from ATCC (Manassas, VA). Medium consisted of DMEM-Ham's F-12 (1:1) supplemented with 4 mM L-glutamine, 10 mM HEPES buffer, 5 pM triiodothyronine, 10 ng/ml recombinant human EGF, 3.5 μg/ml ascorbic acid, 5 μg/ml transferrin, 5 μg/ml insulin, 25 ng/ml prostaglandin E1, 25 ng/ml hydrocortisone, and 8.65 ng/ml sodium selenite (all from Sigma). For RPTEC/TERT1 cells the medium was supplemented with 100 μg/ml G418 (Sigma). Human renal proximal tubular epithelial cells (HTEC) were obtained from patient kidney tissue. All procedures were performed using aseptic techniques. Segments of macroscopically and histologically normal renal cortex (5–10 g) were obtained aseptically from the normal pole of adult human kidneys removed surgically because of small (<6 cm) renal cell carcinomas. Patients were otherwise healthy and not on medication. Informed consent was obtained prior to each operative procedure and the use of human renal tissue for primary culture was reviewed and approved by the local institutional review board at Kyung Hee University Hospital at Gangdong (#2015-04-022, KHNMC). The cells were then cultured in specialized tubular epithelial cell growth media [DMEM-Ham's-F-12; Insulin-Transferrin-Selenium supplements (GIBCO), hydrocortisone (Sigma), MEM Non-Essential amino acids (GIBCO), 10% FBS and 1% P/S]. Primary cultured tubular epithelial cells (PTC) were isolated from the kidneys of WT and *Nlrp3*^−/−^ mice. The cortex was then finely minced and placed in collagenase A (Sigma-Aldrich, St. Louis, MO) and incubated at 37°C for 60 min with frequent shaking. After incubation, the digested mixture was differentially sieved (100–70 um) and washed three times with fresh media. The cells were then cultured in specialized tubular epithelial cell growth media [DMEM-Ham's-F-12 (WelGENE, Daegu, Republic of Korea); Insulin-Transferrin-Selenium supplements (GIBCO), hydrocortisone (Sigma), MEM Non-Essential amino acids (GIBCO), 10% FBS and 1% P/S, to promote growth of tubular epithelial cells but no other cell types. The epithelial phenotype was confirmed to be >95% of cultured cells, using western blot detecting for aquaporin 1 as well as for the proximal tubules in kidney. To maintain the epithelial phenotype, PTC was not used beyond passage 3.

### Masson's trichrome staining

Kidneys were fixed in 10% neutral buffered formalin, embedded in paraffin, cut into 4 μm sections, and stained with Masson's trichrome reagent. Fibrosis was quantified by Masson's trichrome staining and computer-assisted image analysis. Image data was analyzed using Photoshop, version CS6 (Adobe Systems Inc., San Jose, CA). All images were obtained using a 10x objective. Positive blue signal, indicating collagen, was selected based on its color ranges and the proportional area in each image was quantified using the histogram tool and expressed as a percentage of the image.

### TdT mediated dUTP nick end labeling (TUNEL) assay

Apoptosis in renal tissues was identified by TUNEL assay with an *in situ* Cell Death Detection kit following the manufacturer's instruction (Roche Applied Science, Indianapolis, IN). The number of apoptotic cells was counted under fluorescence microscope at ×200 magnification. At least 10 areas at the corticomedullary junction in the sections from different mice of each group were determined and averaged.

### Immunohistochemistry

For immunohistochemical staining, paraffin-embedded kidney sections were deparaffinized, hydrated, and antigen-retrieved, and endogenous peroxidase activity was quenched by 3% H_2_O_2_. Sections were then blocked with 1% bovine serum albumin, followed by incubation with anti-8-OHdG(JAICA) over night at 4°C. After incubation with secondary antibody for 1 h, sections were detected by 3,3′diaminobenzidine (Vector Laboratories, Burlingame, CA) staining. The slides were counterstained with hematoxylin. Images were taken using a Nikon Eclipse 80i microscope (Japan) and Nikon ACT-1 software (Japan). 8-OHdG intensity was scored semi-quantitatively on a scale of 0 = negative, 1 = mild, 2 = moderate, 3 = strong positivity.

### Isolation of mitochondria (MT)

HK-2 cells were harvested from the cultures and fractionated into cytosol (Cyt) and mitochondria by using Mitochondria isolation kit (BioChain Institute, Inc. CA, USA) according to the instruction of the manufacturer. Briefly, Cells are collected and washed with 10 ml ice cold PBS. Centrifuge at 600 g for 5 min at 4°C and resuspend the pellet in 1 ml 1 × Mitochondria Isolation Buffer and transfer into a pre-chilled Dounce tissue grinder. Homogenization was performed on ice and centrifuged for 10 min at 600 g. The collected supernatant was centrifuged for 15 min at 12,000 g. The resulting pellet was resuspended in 100 μl Lysis Buffer with 1 × protease inhibitors.

### Immunocytochemistry

Cells were fixed with 4% paraformaldehyde (Sigma-Aldrich, MO, USA) in PBS for 1 h at room temperature and permeabilized with 1% bovine serum albumin and 0.1% Triton X-100 in PBS. After blocking with 1% bovine serum albumin and 0.1% Triton X-100 in PBS, the slides were incubated with primary antibodies to NLRP3, ASC, MAVS, Mitotracker, and Lysotracker (Molecular Probes, Eugene, OR) overnight at 4°C. Next, the slides were incubated with a FITC-conjugated goat anti-mouse IgG (1:500 dilution; Santa Cruz, CA, USA) for 2 h at room temperature. The slides were mounted with VECTASHIELD HardSet Mounting Medium with DAPI, and images were acquired by a confocal microscope (Carl Zeiss LSM 700, Oberkochen, Germany).

### Measurement of mitochondrial membrane potential (ΔΨ_m_)

HK-2 cells mitochondrial membrane potential ΔΨ_m_ was measured by the sensitive and relatively mitochondrion-specific lipophilic cationic probe fluorochrome 5,5′,6,6′-tetrachloro-1,1′,3,3′-tetraethylbenzimidazoly-carbocyanine iodide (JC-1; Molecular Probes, Eugene, USA). Briefly, HK-2 cells were incubated with JC-1 (5 μmol/L) at 37°C for 20 min and examined by flow cytometry (BD FACSCaliber Flow Cytometry, USA) and confocal microscope.

### Measurement of mitochondrial superoxide generation

Mitochondrial superoxide generation was assessed in live cells with MitoSOX Red (Molecular Probes, Eugene, USA), which is a fluorogenic dye that is taken up by mitochondria, where it is readily oxidized by superoxide. HK-2 cells were incubated with 5μM MitoSOX Red at 37°C for 10 min and examined by flow cytometry (BD FACSCaliber Flow Cytometry, USA) and confocal microscope.

### Annexin V apoptosis assays

To assess the number of apoptotic cells, the FITC Annexin V Apoptosis Detection Kit (BD Biosciences, San Francisco, CA, USA) was used according to the manufacturer's instructions. Briefly, following treatment the cells washed twice cold PBS and the resuspend cells in 1X Binding Buffer at a concentration of 1 × 10^6^ cells/ml. That transfer 100 μl of the solution to a 5 ml culture tube and add 5 μl of FITC Annexin V and 5 μl PI. Gently vortex the cells and incubate for 15 min at RT (25°C) in the dark and add 400 μl of 1X Binding Buffer to each tube. The cells examined by flow cytometry (BD FACSCaliber Flow Cytometry, USA).

### Small interfering RNA knockdown experiments

Duplex small interfering RNAs (siRNAs) targeting NLRP3 (ORIGENE, Rockville, MD, USA) and a control siRNA, siASC, siCaspase-1 and siMAVS were purchased from Bioneer Inc. (Seoul, Korea). HK-2 cells were transfected using Lipofectamine 2,000 (Invitrogen, Carlsbad, CA), after which these cells were utilized for the functional studies 24 to 72 h later, and knockdown efficiency was assessed by Western blot analysis using anti-NLRP3, ASC, caspase-1, MAVS and anti-β-actin antibodies.

### Western blot analysis

Kidneys were washed with PBS and lysed in an ice-cold lysis buffer containing protease inhibitor cocktail (Roche Diagnostics, Mannheim, Germany). Proteins were separated with 10% PAGE and electroblotted onto a PVDF membrane (Millipore, Madrid, Spain). The membrane was incubated with primary antibody raised against NLRP3 (1:1000, AdipoGen, San Diego, USA), ASC, caspase-1, IL-1β, MAVS, LC3, parkin, PINK1, HIF-1α, AIF, cytochrome c, Bax, prohibitin, α-SMA (1:1000, Abcam, Cambridge, UK), GAPDH (1:1000, cell signaling MA, USA), β-actin (1:1000, Santa Cruz, CA, USA) and, subsequently, with horseradish peroxidase-conjugated goat anti-rabbit or mouse immunoglobulin G (1:10000, Santa Cruz, CA, USA). The immunoreactive bands were detected by chemiluminescence (ECL, BioFX Laboratories, Inc. MD, USA). β-actin and GAPDH were used as internal controls of tubular cells and renal tissues. Prohibitin and GAPDH was used for the internal control of mitochondria and cysotol, respectively.

### Statistical analysis

Statistical analyses were conducted using SPSS software (version 20 SPSS, Inc., Chicago, IL). All values are expressed as means ± SE. Results were analyzed using the Kruskal-Wallis non-parametric test for multiple comparisons. Significant differences in the Kruskal-Wallis test were confirmed by the Wilcoxon rank sum and Mann-Whitney test (used to compare mean differences); *P*-values < 0.05 were considered statistically significant.

## Results

### NLRP3 was upregulated and relocalized to mitochondria in renal tubular epithelial cells under hypoxia in the absence of IL-1 β release

**Figure 1:**
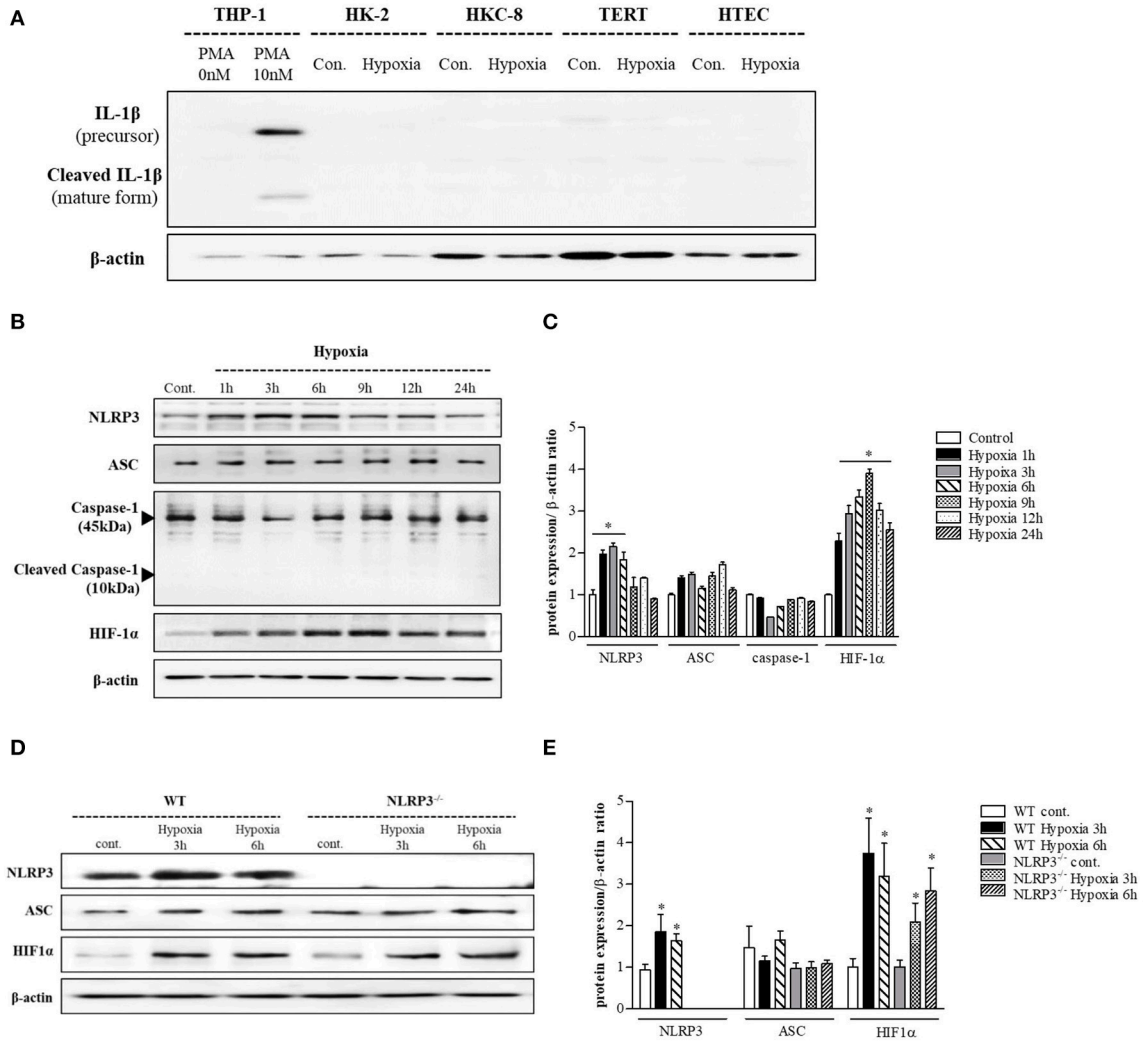
NLRP3 is increased by hypoxia in renal tubular epithelial cells without releasing IL-1 β. **(A)** HK-2, HKC-8, TERT and HTE cells were subjected to 6 h of hypoxia. IL-1β expression was analyzed by immunoblotting. **(B,C)** Expression of NLRP3, ASC, caspase-1, and HIF-1α proteins was detected in HK-2 cells after 0, 1, 3, 6, 9, 12, or 24 h of hypoxia exposure. ^*^p < 0.05 vs. Control. **(D,E)** Expression of NLRP3, ASC, and HIF-1α proteins was detected in PTEC after 3 and 6 h of hypoxia exposure. ^*^p < 0.05 vs. WT control.

**Figure 2:**
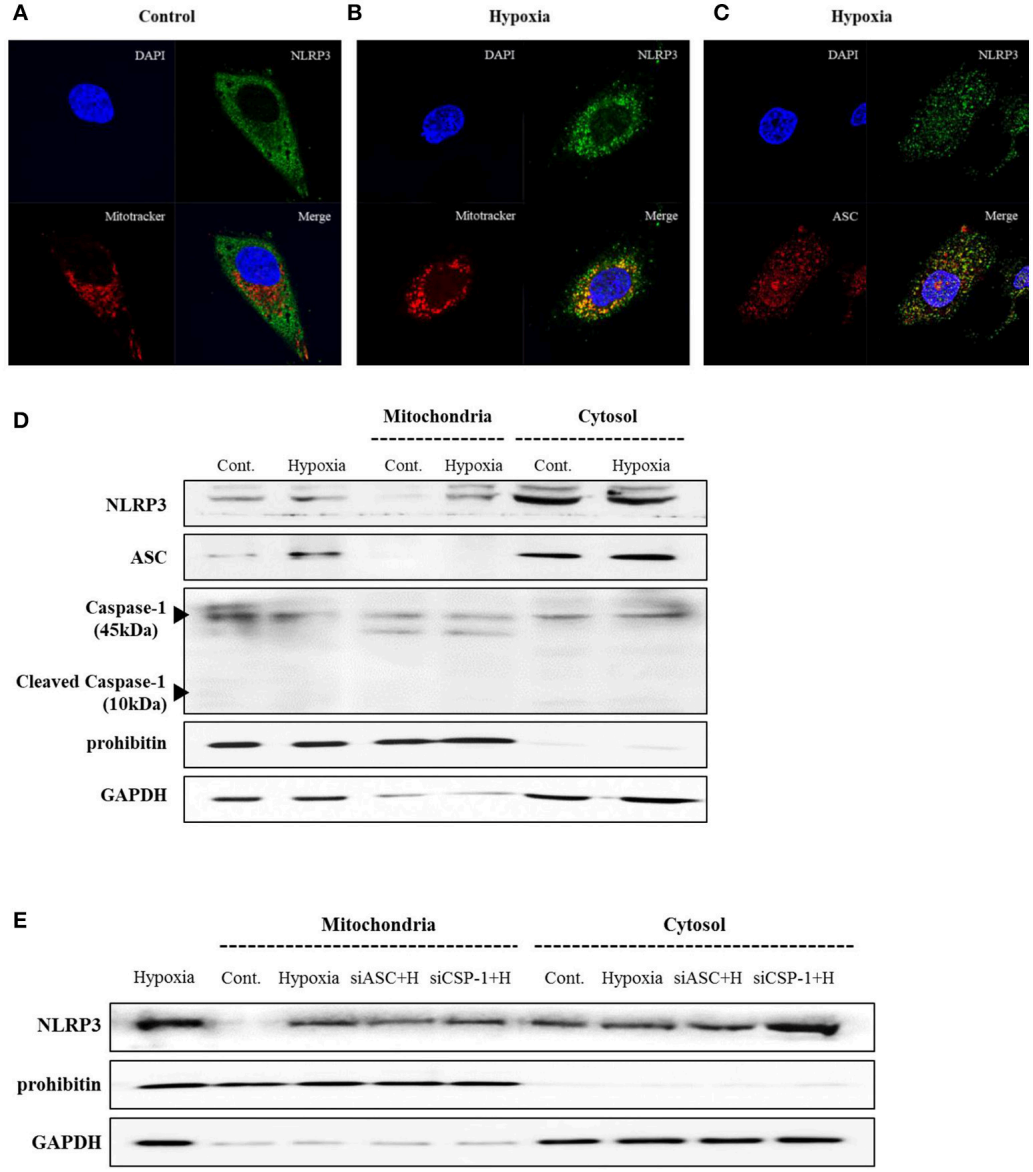
NLRP3 relocalizes into the mitochondria during hypoxia in HK-2 cells. **(A**–**C)** HK-2 cells were subjected to 6 h of hypoxia and analyzed by confocal microscopy for NLRP3, ASC and MitoTracker staining. **(D)** Expression of NLRP3, ASC and Caspase-1 was analyzed by immunoblotting in hypoxia-exposed cells. **(E)** HK-2 cells were transfected with siASC and siCaspase-1 and subjected to 6 h of hypoxia. NLRP3 expression was analyzed by immunoblotting. Cell lysates were fractionated into cytosolic or mitochondrial fraction. Prohibitin and GAPDH were used to confirm that the mitochondrial and cytosolic fractions were well separated.

THP-1 cells expressed pro-IL-1β and released of IL-1β in response to phorbol myristoyl acetate (PMA) stimulation, however none of the renal tubular epithelial cells including HK-2, HKC-8, TERT, and HTEC expressed IL-1β under hypoxic conditions (Figure 1A). NLRP3 expression was increased until 6 h of hypoxic stimuli and then decreased in HK-2 cells; however, ASC and pro-caspase-1 levels did not in response to hypoxia (Figures 1B,C). ASC expression in proximal tubular epithelial cells (PTEC) from NLRP3 KO mice did not change under hypoxic injury despite an increase in HIF-1α expression (Figures 1D,E). These results indicate that NLRP3 expression was increased in renal tubular epithelial cells in hypoxia independent of inflammasome. NLRP3 is located in the cytoplasm of tubular cells, but, it relocalizes to the mitochondria under hypoxic conditions. Confocal microscopy demonstrated that NLRP3 was localized in the cytoplasm in normoxia but colocalized with the mitochondrial marker, MitoTracker, after 6 h of hypoxia (Figures 2A,B). ASC did not significantly colocalize with NLRP3 significantly under hypoxia (Figure 2C). Only NLRP3 was present in the mitochondrial fraction under hypoxia in HK-2 cells (Figure 2D). This relocalization of NLRP3 to the mitochondria was not influenced by the deletion of other inflammasome components, ASC or caspase-1 (Figure 2E).

### Deletion of NLRP3 ameliorated mitochondrial damage and apoptosis in hypoxia

**Figure 3:**
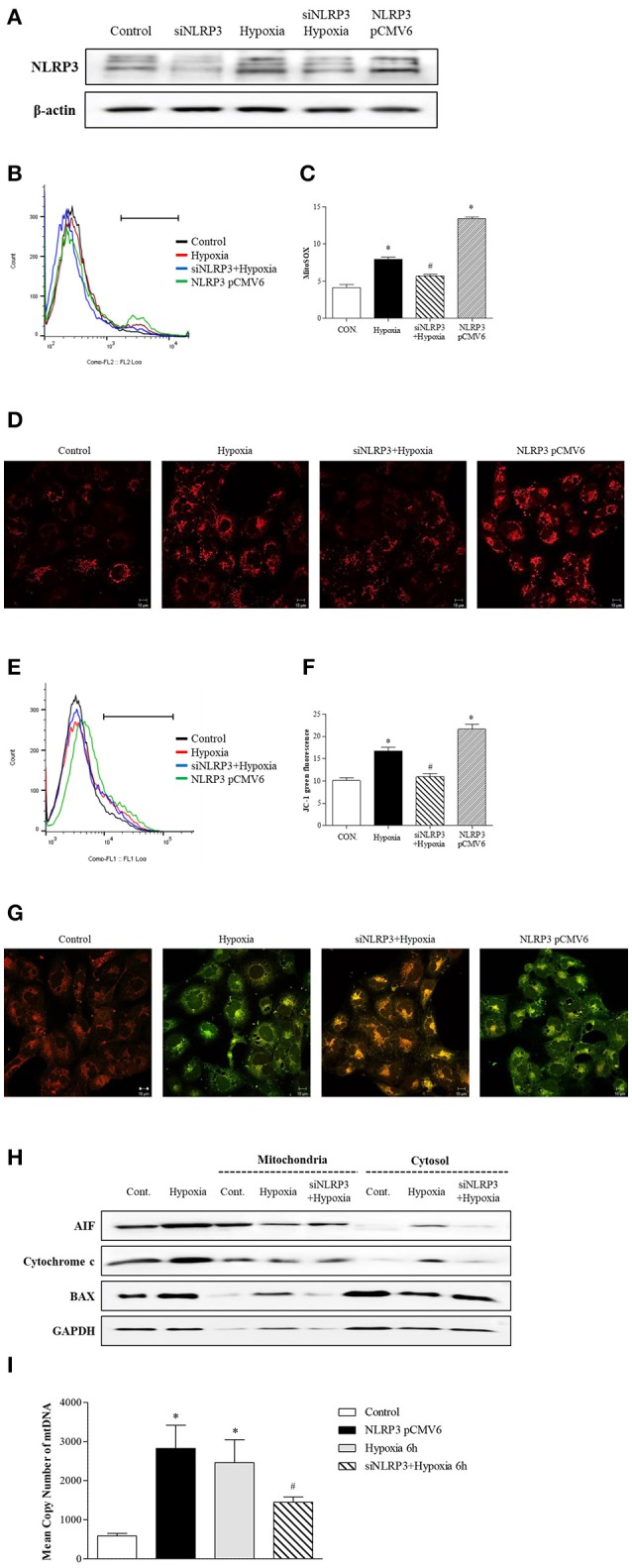
NLRP3 is required for mitochondrial dysfunction in HK-2 cells. **(A)** HK-2 cells were transfected with siNLRP3 and NLRP3 pCMV6 vector and subjected to 6 h of hypoxia. NLRP3 expression was analyzed by immunoblotting. **(B**–**D)** siNLRP3 or NLRP3 pCMV6 transfected HK-2 cells were stained with MitoSOX and analyzed by flow cytometry and confocal microscopy.**(E–G)** siNLRP3 or NLRP3 pCMV6 transfected HK-2 cells were stained with JC-1 and analyzed by flow cytometry and confocal microscopy. Representative histograms and quantified levels are shown. ^*^p < 0.05 vs. Control, #*p* < 0.05 vs. Hypoxia. **(H)** HK-2 cells were transfected with siNLRP3 and subjected to 6 h of hypoxia. Cell lysates were fractionated into cytosolic or mitochondrial fraction and then immunoblotted for AIF, cytochrome c, and Bax. **(I)** HK-2 cells were transfected with siNLRP3 and NLRP3 pCMV6 vector; and subjected to 6 h of hypoxia. Genomic DNA and cytosolic mtDNA were isolated from cells and mtDNA in cytosolic fraction was analyzed by real-time PCR. ^*^p < 0.05 vs. Control, #*p* < 0.05 vs. Hypoxia.

To clarity the effect of inflammasome-independent NLRP3 on the mitochondria in hypoxia, we evaluated whether the deletion of NLRP3 could affect mitochondrial injury. Small interfering (si) RNA and pCMV6 were transfected into HK-2 cells to delete and increase of NLRP3 expression, and the efficiency was tested by western blotting (Figure 3A). Flow cytometry and confocal microscopy with MitoSOX^TM^ Red were used to detect mitochondrial superoxide levels. Hypoxia increased mitochondrial superoxide in tubular epithelial cells, and this increase was blocked by NLRP3 siRNA (Figures 3B–D). Overexpression of NLRP3 was sufficient to increase the level of mitochondrial superoxide in normoxia condition. To evaluate whether the increased production of mitochondrial superoxide by hypoxia leads to mitochondrial dysfunction, we confirmed the effect of hypoxia on mitochondrial membrane potential by JC-1 staining. Depolarized regions of mitochondria are indicated by the green fluorescence of JC-1 monomers. Hypoxia induced the depolarization of the mitochondrial membrane, resulting in an increase in green fluorescence (and a decrease in orange fluorescence); this hypoxia-induced depolarization was rescued by NLRP3 siRNA (Figures 3E–G). Depolarization of mitochondria was markedly observed in NLRP3 overexpressed HK-2 cell without hypoxia. The effects of NLRP3 inhibition on the mitochondria attenuated the release of pro-apoptotic proteins from the mitochondrial intermembrane space. Hypoxia induced the release of apoptosis-inducing factor (AIF) and cytochrome c into the cytosolic fraction and translocation of Bax. These effects were significantly attenuated by NLRP3 deletion (Figure 3H). We further confirmed the role of NLRP3 in the mitochondrial damage under hypoxia through the deletion and overexpression of NLRP3 in tubular cells. The overexrpression of NLRP3 in tubular epithelial cells by pCMV6 transfection resulted in a marked increase in the copy number of mitochondrial DNA (mtDNA) in the cytosol even in normoxia (Figure 3I). Furthermore, the deletion of NLRP3 was protective against mitochondrial injury under hypoxia.

### Mitochondrial antiviral signal protein (MAVS) is a critical binding partner of NLRP3 in the mitochondria

**Figure 4:**
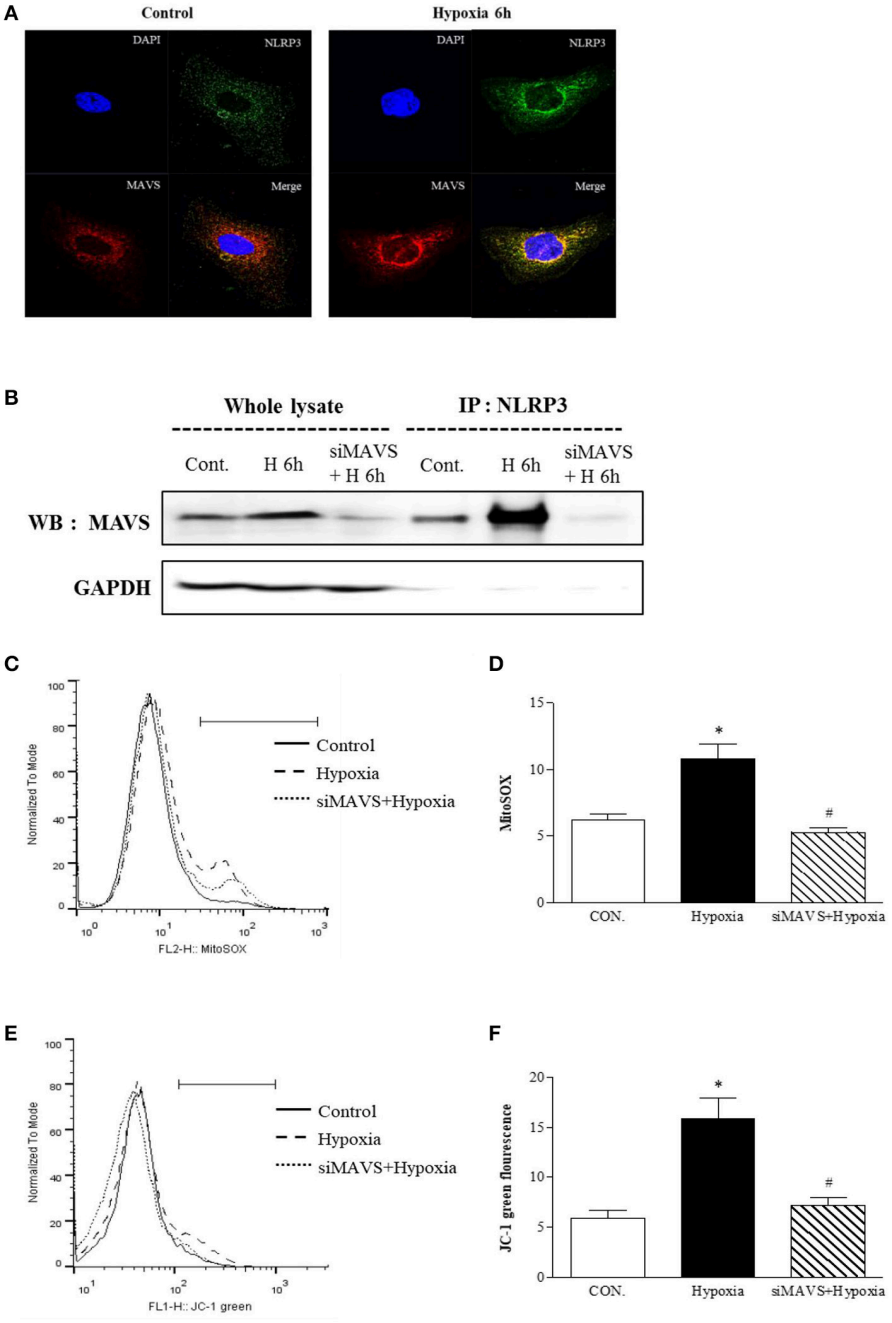
The mitochondrial adaptor MAVS mediates NLRP3 mitochondrial localization. **(A)** Hypoxia-exposed HK-2 cells were analyzed by confocal microscopy for NLRP3 and MAVS expression. **(B)** HK-2 cells were transfected with siMAVS and subjected to 6 h of hypoxia. Cell lysates were immunoprecipitated using anti-NLRP3 antibody, and the immunoprecipitates were immunoblotted with anti-MAVS antibody. **(C,D)** HK-2 cells were transfected with siMAVS and subjected to 6 h hypoxia. HK-2 cells were stained MitoSOX and analyzed by flow cytometry. **(E,F)** HK-2 cells were transfected with siMAVS and subjected to 6 h of hypoxia. HK-2 cells were stained with JC-1 and analyzed by flow cytometry. Representative histograms and quantified levels are shown. ^*^p < 0.05 vs. Control, #*p* < 0.05 vs. Hypoxia.

Since our findings showed that inflammasome-independent NLRP3 is critical for mitochondrial function, we designed an experiment to examine whether inflammasome-independent NLRP3 could colocalize and interact with MAVS under hypoxia. After 6 h of hypoxia, relocalized NLRP3 to the mitochondria and colocalized with MAVS (Figure 4A). Immunoprecipitation of NLRP3 in tubular epithelial cells demonstrated that NLRP3 interacts specifically with the MAVS under hypoxia (Figure 4B). We examined whether the knockdown of MAVS by siRNA transfection might reduce the NLRP3-induced mitochondrial superoxide production and depolarization of the mitochondrial membrane potential under hypoxia (Figures 4C–F). Indeed, the increase in MitoSOX and JC-1 fluorescence by hypoxia was attenuated by MAVS siRNA, and these effects were similar to NLRP3 deletion. These results indicate that the interaction of NLRP3 with MAVS is a major activator of inflammasome-independent NLRP3-induced mitochondrial dysfunction in hypoxia.

### NLRP3 deletion protects to hypoxia-induced mitochondrial injury and apoptosis

**Figure 5:**
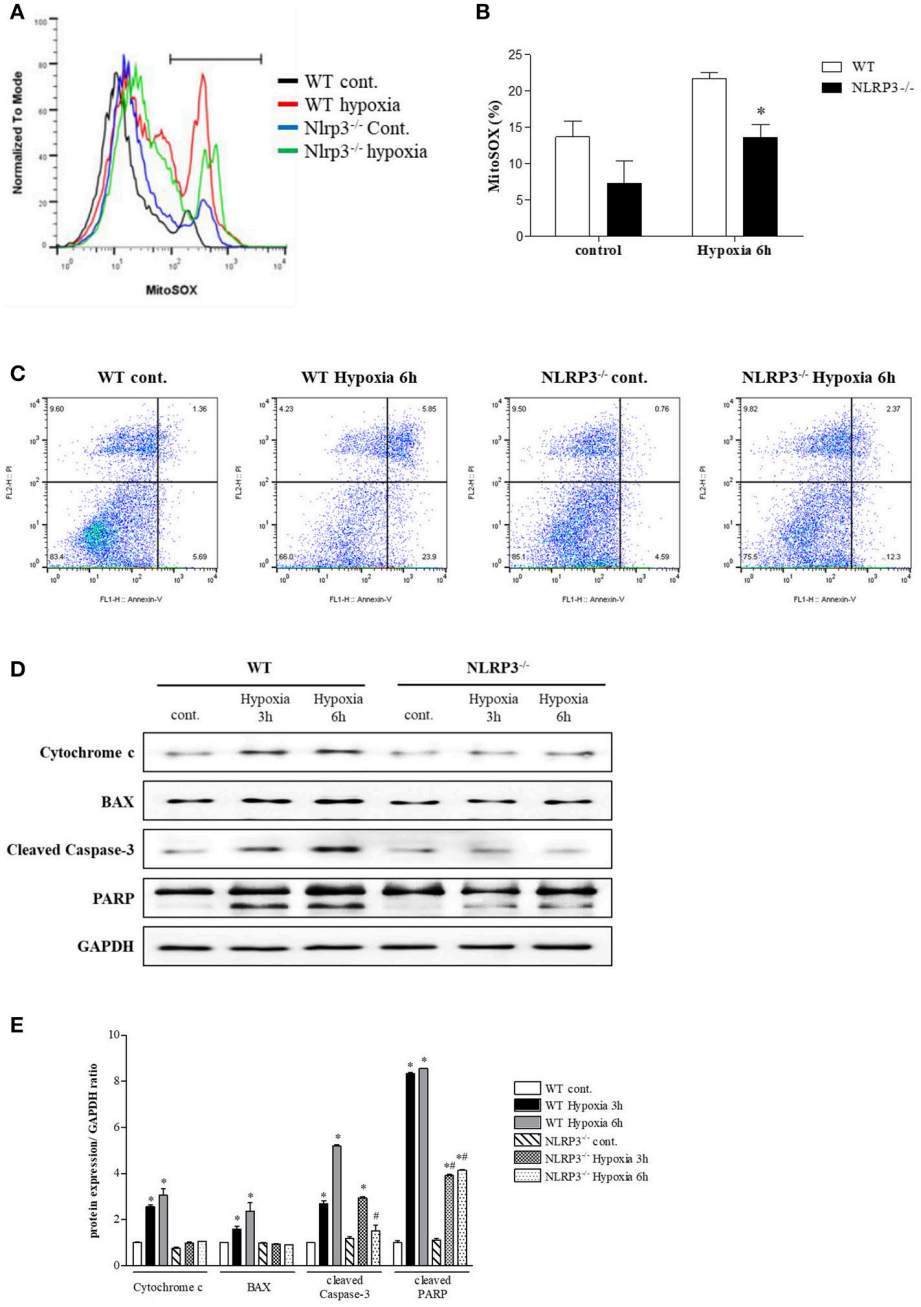
NLRP3 deficiency in PTEC inhibits hypoxia-induced mitochondrial ROS and apoptosis. **(A,B)** PTEC were stained with MitoSOX and analyzed by flow cytometry. Representative images and quantitative analysis of mean fluorescence intensities are shown. ^*^p < 0.05 vs. WT. **(C)** Analysis of the cell death phenotype by Annexin-V/PI staining followed by flow cytometry. **(D,E)** Expression of cytochrome c, Bax, cleaved caspase-3 and PARP proteins was detected in WT and NLRP3 KO PTEC after 3 and 6 h of hypoxia exposure. ^*^p < 0.05 vs. WT control, #*p* < 0.05 vs. WT hypoxia. NLRP3^−/−^, NLRP3 KO.

To assess the protective role of NLRP3 inhibition against hypoxia-induced mitochondrial damage, we subjected PTEC from WT and NLRP3 KO mice to hypoxic injury. Compared with that in WT mice, hypoxia-induced mitochondrial superoxide generation was attenuated in PTEC from NLRP3 KO (Figures 5A,B). We investigated apoptosis patterns by staining the cells with Annexin-V, which binds to phosphatidylserine, and propidium iodide (PI), a membrane-impermeable DNA stain. After 6 h of hypoxia exposure, the majority of PTEC from WT mice lost membrane integrity and became PI-positive (23.9%). In contrast, PTEC from NLRP3 KO were resistant to hypoxic injury and displayed decreased proportions of cells in early and late apoptosis, both (2.4 and 12.3%, respectively, Figure 5C). We further confirmed the release of mitochondrial pro-apoptotic and apoptotic molecules. The increased in cytochrome c, Bax, cleaved caspase-8, cleaved caspase-3 and poly (ADP-ribose) polymerase (PARP) expression in PTEC from WT under hypoxia was ameliorated in PTEC from NLRP3 KO mice (Figures 5D,E).

### NLRP3 deletion attenuates renal injury by preventing apoptosis

**Figure 6:**
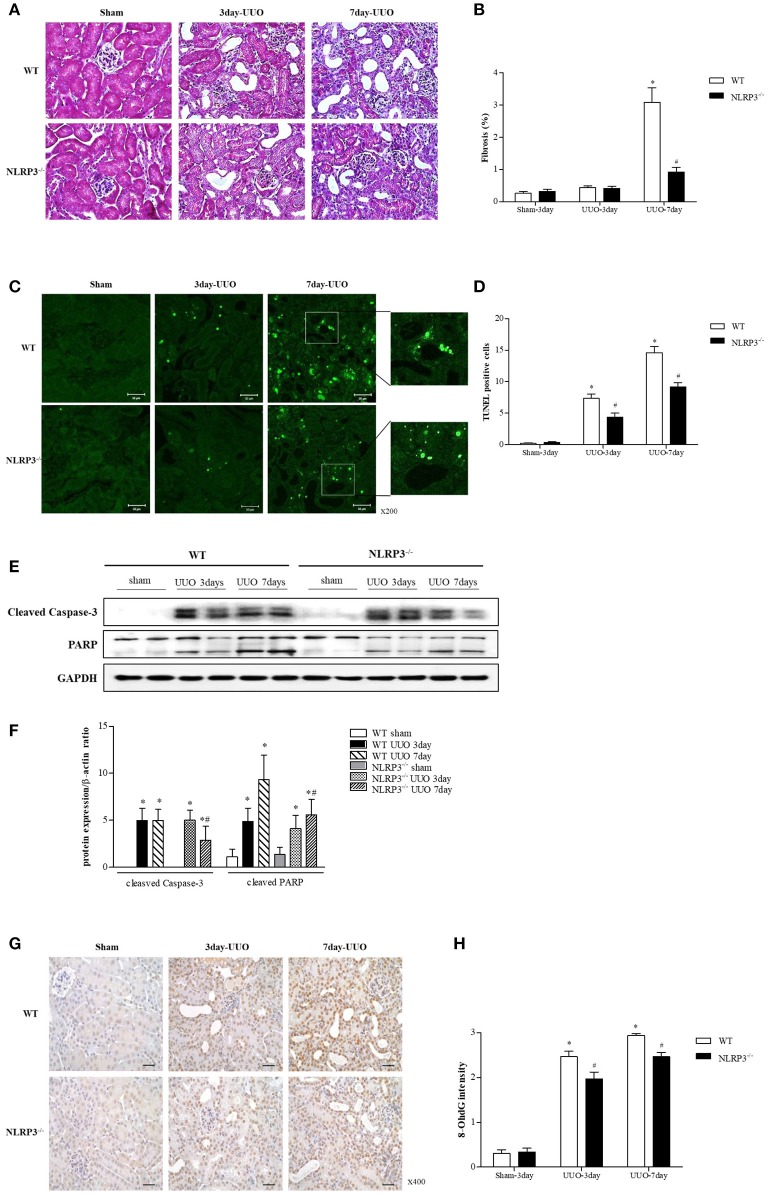
NLRP3 contributes to tubular injury in mice undergoing UUO. **(A,B)** Masson's trichrome–stained of kidney sections from WT (C57BL/6) mice and NLRP3 KO mice after 3 and 7 days of UUO (Original magnification, x400). Semiquantitative scoring of tubulointerstitial fibrosis in Masson's trichrome–stained sections. **(C,D)** Representative images of TUNEL staining are shown (Original magnification, ×200). Bar graph indicates the mean number of TUNEL-positive tubular cells per field. **(E,F)** Expression of caspase-3 and PARP proteins was detected in kidney tissue samples. **(G,H)** Immunohistochemistry was used to measure the levels of the oxidative stress marker 8-OHdG (Original magnification, ×400). Bar graph indicates the mean number of 8-OHdG-positive cells. ^*^p < 0.05 vs. WT sham, #*p* < 0.05 vs. WT UUO.

We further confirmed the protective role of inflammasome-independent NLRP3 in tubular epithelial cells associated with apoptosis *in vivo* using a unilateral ureteral obstruction (UUO) model. Interstitial fibrosis in obstructed kidney was detected by Masson's trichrome staining at 3 and 7 days after UUO. Renal fibrosis significantly decreased after UUO in the kidney of NLRP3 KO mice (Figures 6A,B). There was no change in BUN and serum creatinine in NLRP3 KO mice compared with that in WT mice (data was not shown). The number of TUNEL-positive tubular epithelial cells was markedly increased after UUO in WT kidney compared with that in NLRP3 KO kidney on both days 3 and 7, both (Figures 6C,D). The *in vivo* effects of NLRP3 on apoptosis were confirmed by cleaved caspase-3 and PARP expression in the kidney tissues. The expression of cleaved caspase-3 and PARP was significantly increased after UUO in WT mice, and this increase was alleviated in the NLRP3 KO mice on days 3 and 7 after UUO (Figures 6E,F). Oxidative stress was also attenuated in NLRP3 KO kidney. After UUO, immunohistochemistry staining intensity for the marker of DNA oxidative damage, 8-hydroxy-2′-deoxyguanosine (8-OHdG), was significantly attenuated on both 3 and 7 days in NLRP3 KO mice relative to that in WT mice (Figures 6G,H).

### Effects of NLRP3 on mitophagy

**Figure 7:**
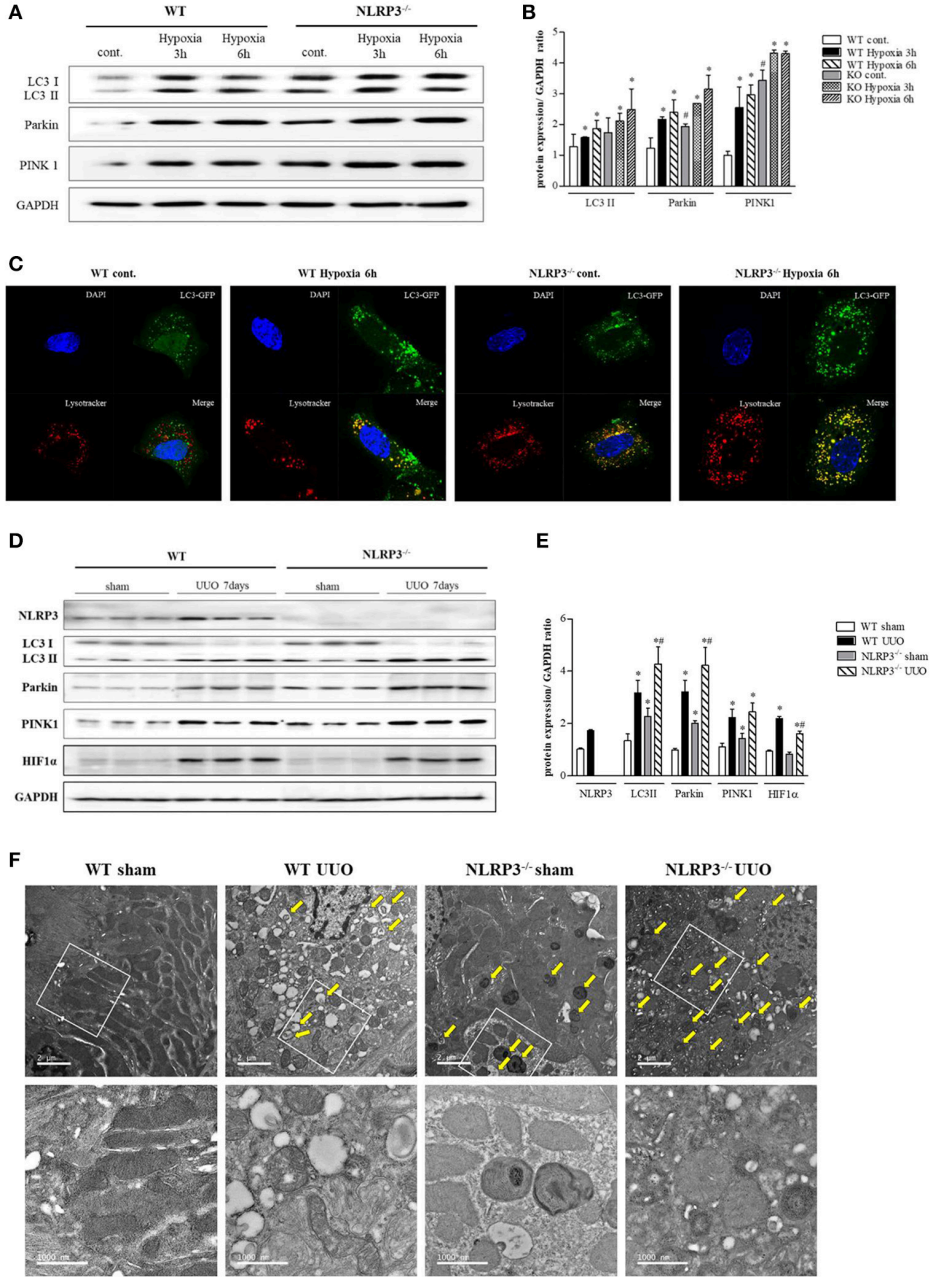
NLRP3 depletion is associated with mitophagy, which protects mitochondria against damage. **(A,B)** Expression of LC3, Parkin, and PINK1 proteins was detected in WT and NLRP3 KO PTEC after 3 and 6 h of hypoxia exposure. ^*^p < 0.05 vs. WT control, #*p* < 0.05 vs. WT hypoxia. **(C)** PTEC were transfected with LC3-GFP vector and subjected to 6 h of hypoxia. Hypoxia-exposed PTEC were stained with Lysotracker-Red and analyzed by confocal microscopy. **(D,E)** Expression of NLRP3, LC3, Parkin, PINK1 and HIF-1α proteins was detected in kidney tissue samples. ^*^p < 0.05 vs. WT sham, #*p* < 0.05 vs. WT UUO. **(F)** Transmission electron microscopy analysis of mitochondrial morphology. Arrows indicate mitophagy. Original magnification × 1,000 nm.

Further experiment were performed to clarity how NLRP3 depletion could protect cells against mitochondrial injury. Mitophagy is critical for appropriate cellular functions, as it eliminates damaged mitochondria. We assessed mitophagy in tubular epithelial cells and kidney tissues. LC3 II, parkin, and PTEN-induced putative kinase 1 (PINK1) were all upregulated relative to baseline in tubular cells of NLRP3 KO mice (Figures 7A,B). GFP-LC3 punctate that colocalizes with lysosome is widely used as markers of autophagy. We evaluated the activity of autophagosomes in tubular epithelial cells from NLRP3 KO mice and observed higher expression under hypoxia than in cells from WT (Figure 7C). These augmented mitophagy activities in tubular epithelial cells of NLRP3 KO mice were similarly observed in the UUO model. NLRP3 KO mice displayed higher expression of LC3 II, parkin and PINK1 in both sham and UUO-treated kidneys than did WT mice (Figures 7D,E). We further investigated the morphology of mitochondria and mitophagosomes in kidney tissues of WT and NLRP3 KO. Compared with that in WT kidneys, the number of mitophagosomes was increased in NLRP3 KO kidneys (Figure 7F). After UUO, vacuolization, crista disruption, and disappearance of normal morphology of mitochondria were increased in both WT and NLRP3 KO mice but the number mitophagosomes was higher in NLRP3 KO mice kidney than in WT kidney.

## Discussion

In the present study, we demonstrated that inflammasome-independent NLRP3 in renal tubular epithelial cells regulates mitochondrial damage and apoptosis through MAVS under hypoxic injury. The regulation of NLRP3 in renal tubular cells resulted in changing of mitochondrial ROS production and mitochondrial membrane potentials. The deletion of NLRP3 attenuates apoptosis, fibrosis, and ROS injury after UUO compared with that of WT mice. These results suggest that inflammasome-independent NLRP3 in renal tubular cells could be evaluated more as a treatment target of AKI.

Inflammasome-independent NLRP3 in renal tubular epithelial cells increased during early hypoxia and relocalized with MAVS. MAVS is known to mediate NF-κB and type I interferon signaling during infection with RNA viruses and require for the recruitment and optimal activation of the NLRP3 inflammasome (20–23). MAVS-depleted immune cells fail to secrete IL-1β after stimulation with various inflammasome activators, and MAV is associated with ASC oligomerization for efficient assembly of the NLRP3 inflammasome (24). However, there is no clear evidence of whether MAVS is an essential binding partner of NLRP3 in non-immune cells. One study revealed that NLRP3 colocalized with the mitochondria in HEK 293T cells overexpressing NLRP3 and nearly disappeared in MAVS siRNA-treated cells (22). Our results firstly identified MAVS as an indispensible molecule for inflammasome-independent NLRP3 activation because mitochondrial ROS production and depolarization of mitochondrial membrane were attenuated by MAVS deletion and since MAVS deletion was as effective as NLRP3 deletion in renal tubular epithelial cells. Recent studies have identified some mitochondria-related proteins interacted with MAVS signaling and mediated mitochondrial homeostasis(8, 25). Also the activation of MAVS was linked to inducing autophagy by interacting with LC3 protein and MAVS had a potential receptor of mitophagy(26). Further study about the MAVS as a possible modulator on mitophagy should be needed.

Many studies have reported on the important role of the NLRP3 inflammasome in diabetic nephropathy, hypertensive kidney disease, rhabdomyolysis, UUO and ischemic renal disease (1, 23, 27–29). These studies demonstrated that the genetic deletion of NLRP3 dampened the progression of renal disease, but did not differentiate whether the protective effect originates from the depletion of inflammasome-dependent NLRP3 in innate immune cells or inflammasome-independent NLRP3 in resident renal cells. Some studies have suggested the possibility that NLRP3 deletion in renal epithelial cells could be more important than that in bone marrow-derived cells, but the exact role of NLRP3 in non-immune cells has not been evaluated in detail (1, 12). Interestingly, renal injury following IRI was improved in NLRP3 KO mice but not reversed by the blockade of IL-1 with anakinra or the deletion of IL-1R or IL-18 (12). Our results demonstrated that various types of renal tubular epithelial cells such as HK-2 cell, HKC-8 cell, TERT cell and HTEC did not secrete pre-IL-1β and active 1β under hypoxic condition. Nevertheless renal tubular cells contained considerable amount of the NLRP3 protein even in unstimulated condition and it increased in hypoxic circumstance. Considering these results, we could speculate that the NLRP3 in renal tubular cells should act as an inflammasome-independent protein, unlike immune cells. We used HIF-1α as a hypoxic marker in this study. However, recent study introduced HIF-1α as a possible regulator to activate NLRP3 inflammasome in venous thrombosis(30). Several damage associated with molecular patterns (DAMPs) such as ATP, uric acid, and ROS has been known to activate NLRP3 inflammasome, however the precise mechanism how these DAMPs initiated to stimulate NLRP3 inflammasome has not been clarified (31). Further study should be needed to identify the HIF-1α as a potential agonist of NLRP3. NLRP3-depletion in tubular epithelial cells attenuated the increase in mitochondrial ROS production and depolarization of the mitochondrial membrane and finally contributed to the decrease in cell death. Studies on NLRP3 signaling have consistently described its role of the mitochondria in immune cells with an inflammasome-dependent manner (32). However, evidence support that inflammasome-independent NLRP3 regulates mitochondrial ROS in cardiac fibroblast under TGF-β1 stimulation (33). Our findings suggest the possibility that mitochondrial injury in non-immune cell could be controlled by the regulation of inflammasome-independent NLRP3.

Renal tubular epithelial cells are the main site of injury in various kinds of kidney disease, especially in AKI. Apoptosis of renal tubular cells after AKI is the major initial step in the progression of AKI to CKD associated with tubulointerstitial fibrosis (25, 26). Based on these evidences, many therapeutic treatments in AKI have focused on decreasing apoptosis in tubular epithelial cells at the onset of injury. Controlling apoptosis is also important as cell death will lead to the release of DAMPs, leading to the induction of further inflammation (34). We designed cell experiments to identify the role of NLRP3 not in ischemia-reperfusion circumstance, but in hypoxia condition. UUO injured kidney underwent the ischemic change in early phase after surgery(34, 35). Up-regulated HIF-1α in obstructed kidney revealed renal tissue underwent hypoxic injury. Therefore, we selected the UUO model to ascertain early tubular injury on 3 days and fibrotic change on 7 days after obstructing operation. UUO is the unique model that can be used to evaluate how AKI contributes to tubulointerstitial fibrosis and CKD progression. The present study showed that the prevention of early apoptosis after UUO in NLRP3 KO mice ameliorated tubulointerstitial fibrosis relative to that in WT mice. Ample evidences have indicated that apoptosis is associated with inflammasome-dependent NLRP3. Apoptotic and necrotic cell death machineries both directly and indirectly stimulate inflammasome assembly and IL-1β secretion. However, in contrast with that ASC KO and caspase-1 KO, tubular necrosis after bilateral IRI is significantly attenuated only with NLRP3 KO (8). Finally, we suggest that the regulation of inflammasome-independent NLRP3 in renal tubular epithelial cells could prevent apoptosis in AKI and attenuate tubulointerstitial fibrosis.

We also identified mitophagy as a factor to protect cells against mitochondrial injury in response to hypoxia in inflammasome-independent NLRP3. Hypoxic stress in renal tubular epithelial cells induced autophagy before apoptosis (36). Autophagy inhibition by pharmacologic intervention increased apoptosis in rat kidney proximal tubular cells under hypoxia and worsened renal function and histologic changes. Our study demonstrated that NLRP3-deficient cell and kidney tissues expressed upregulated basal levels of LC3-II, PINK1 and activation of mitophagosomes. The extent of mitophagy was also higher in NLRP3 KO tubular epithelial cells and kidney after UUO than in WT. We suggest that the upregulated mitophagy in NLRP3 KO mice might have a protective role against mitochondrial injury and cell death in tubular epithelial cells, which contribute to decrease tubular apoptosis and fibrosis after AKI. Mitophagy can rapidly remove damaged mitochondria and thus maintain cellular function and dampen additional damages. Previous UUO experiments have revealed that renal fibrosis, inflammation and mitochondrial dysfunction were attenuated in the UUO-treated kidney of NLRP3 KO mice (28, 37). Both of inflammasome dependent and independent NLRP3 is essential for the renal damage in UUO model (28). NLRP3 activation induced mitochondrial damage, and the damaged mitochondria were removed by mitophagy-mediated clearance, thus mitophagy can control excessive inflammation and prevent cell death in immune cells (38). Almost all studies have demonstrated autophagy/mitophagy in immune cells to be dependent on the inflammasome pathway. One study reported that NLRP3-depleted murine lung endothelial cells displayed higher LC3B-II/I ratio and PINK1 expression relative to baseline than did WT cells. Up-regulated autophagy in NLRP3-deficient cells and NLRP3 KO lung tissues was protective against apoptosis during hyperoxia (39). The study speculated that PINK1 mediates the protective effect of NLRP3 depletion by optimizing PGC-1α and parkin interactions, promoting the removal of dysfunctional mitochondria, and maintaining mitochondrial biogenesis in hyperoxia. Therefore, this protective effect could be associated with infammasome-independent NLRP3 in non-immune cells because PTEC from NLRP3 KO mice display already higher expression of LC3B-II/I ratio, PINK1, and parkin from the normoxia. These effects of infammasome-independent NLRP3 in non-immune cells should be evaluated more as an autophagy regulator.

Taken together, our results indicate that inflammasome-independent NLRP3 in renal tubular cells plays an important role in mitochondrial ROS production and injury by relocalizing with MAVS after hypoxic injury. This mitochondrial regulation in the absence of NLRP3 results increases autophagy and attenuates apoptosis after UUO. We thus suggest that infammasome-independent NLRP3 in the kidney could be a therapeutic target of AKI to prevent its progression to CKD.

## Author contributions

S-HL, J-YM, and YK provided idea. YK and S-HL conceived funding and wrote manuscript. S-MK performed cellular experiments and analyzed the results. SL interpreted pathologic findings. YHL, D-JK, and SP performed animal experiments and involved statistical analysis. K-HJ helped to draft the manuscript.

### Conflict of interest statement

The authors declare that the research was conducted in the absence of any commercial or financial relationships that could be construed as a potential conflict of interest.
